# Do More Recent Born Generations of Older Adults Have Stronger Grip? A Comparison of Three Cohorts of 66- to 84-Year-Olds in the Tromsø Study

**DOI:** 10.1093/gerona/gly234

**Published:** 2018-10-11

**Authors:** Bjørn Heine Strand, Astrid Bergland, Lone Jørgensen, Henrik Schirmer, Nina Emaus, Rachel Cooper

**Affiliations:** 1 Department of Chronic Diseases and Ageing, Norwegian Institute of Public Health (NIPH), Oslo; 2 Department of Physiotherapy, OsloMet, Oslo Metropolitan University; 3 Department of Health and Care Sciences, UiT The Arctic University of Norway, Tromsø; 4 Department of Clinical Therapeutic Services, University Hospital of Northern Norway, Tromsø; 5 Department of Clinical Medicine, University Hospital of Northern Norway, Tromsø; 6 Division of Medicine and Laboratory Sciences, Akershus University Hospital, Lørenskog, Norway; 7 MRC Unit for Lifelong Health and Ageing at UCL, UK

**Keywords:** Grip strength, Physical capability, Aging, Secular trends, Birth cohorts

## Abstract

**Background:**

Evidence pertaining to whether more recent born generations of adults reaching old age have better physical capability than previous generations is scarce and inconclusive. We aimed to investigate birth cohort differences in grip strength.

**Methods:**

The study comprised 5,595 individuals from the Tromsø study waves in 1994/1995, 2007/2008, and 2015/2016. Grip strength (bar) was measured using a Martin vigorimeter, and compared across three birth cohorts of 66- to 84-year-olds (born in: 1910–1929, 1923–1942, 1931–1949), as well as within narrower age bands to ensure nonoverlapping cohorts. Linear regression was applied, adjusted for age, education, smoking, physical activity, height, and weight.

**Results:**

Grip strength increased across birth cohorts, and the increase was similar within narrower age bands and across genders. Overall, the increase in sex-adjusted mean grip strength when comparing the first and latest born cohorts, born 21 years apart, was 0.06 bar (95% CI 0.04, 0.07). Higher educational levels, and greater height and weight in the most recent born cohort explained 48% of this difference, while reduced smoking and physical inactivity in more recent born cohorts had little impact.

**Conclusions:**

Our findings suggest higher grip strength in more recent birth cohorts of older Norwegian adults, which can be partly attributed to higher education and greater height. This difference corresponded to a 5-year difference in grip strength; more recent born generations of 80-year-olds, therefore, have similar mean grip strength as 75-year-olds born one generation earlier.

The world population is aging rapidly, but evidence pertaining to whether generations of adults now reaching old age have better health and capability than previous generations is scarce and inconclusive (1,2). Disability, resulting from the loss of physical and cognitive capability, carries high costs to individuals, their families, and society. Unfortunately, whether observed extensions in average life expectancy are accompanied by extensions in disability-free life expectancy remains unclear. It is therefore not currently possible to provide policymakers with the robust empirical evidence they need, for future planning of health and social care provision, on the question of whether people are living healthier, independent lives for longer or are living for extended periods with reduced capability and dependence (3).

One of the challenges of addressing this question is that whether or not evidence is found of improvements in health and capability in more recent birth cohorts, depends on the outcome measure investigated. For example, in studies focusing on disease status, results have generally shown that onset of disease is occurring at the same average ages today as in previous generations (4,5). With increased life expectancy, this implies that people are now living longer with disease prior to death. Findings are more promising in studies of cognitive capability, which have shown consistent evidence of improvement in more recent born cohorts (6–8). However, for self-reported physical disability, results are much more equivocal, vary by country and are heavily dependent on the measures of disability used (8–11). Further, where positive trends in self-reported physical disability have been observed it has been suggested that this may be due to improvements in the environment or to changing perceptions and societal norms related to what constitutes dependence and disability, rather than improvements in intrinsic capacity (12).

To assess this, performance-based measures of physical capability, such as grip strength, which provide an indication of intrinsic capacity may play a critical role (3). This is especially as epidemiological studies have shown that stronger grip from at least as early as midlife onwards is related to lower subsequent risk of disability (13,14) as well as reduced risk of morbidity and premature mortality (15–21), suggesting that it could act as a useful marker of healthy aging (22), and be a feasible prognostic tool in clinical assessment (23).

However, there are a limited number of studies assessing cohort differences in grip strength, or other performance-based measures of physical capability, and findings from these are not as promising as those for cognitive capability (8,9). For example, no improvements in physical capability (as indicated by grip strength, chair stands, and walking speed) were found in a comparison of two Danish cohorts of 93- and 95-year-olds born 10 years apart (8). In the Swedish SWEOLD study, there was evidence that physical capability deteriorated in more recent born cohorts of adults above 76 years over the period 1992 to 2002 (9). Similarly concerning results were reported in a Chinese study of individuals aged 80 and above, where mean levels of performance in a number of objective physical tests (ie, standing up from a chair, picking up a book from the floor, and turning around 360°) deteriorated between 1998 and 2008 (11). A key limitation of these existing studies, for future planning, is their focus on the oldest old born in the first few decades of the 20th century. Whether similar trends are observed at younger ages in more recent born cohorts thus needs to be investigated, especially given major secular trends in key risk factors for disability and physical decline, such as education, smoking and obesity across the 20th century (24).

In this article, we aimed to investigate if there were birth cohort differences in grip strength among Norwegian adults aged 66 to 84 years. If cohort differences were found, we then aimed to examine the extent to which these could be attributed to any observed cohort differences in education, height, weight, leisure time physical activity, and smoking.

## Method

### Study Sample

This study utilizes data from the three Tromsø study waves initiated in 1994, 2007, and 2015, with measures of grip strength. The Tromsø Study is a multipurpose population-based health examination study, initiated in 1974 with study waves repeated in 1979, 1986, 1994, 2001, 2007, and 2015 (25). In the current analyses, the sample was restricted to those aged 66–84 years at one of the three waves in 1994, 2007, or 2015; the age band assessed in all study waves.

The three study waves had slightly different sampling strategies, but response rates remained high and comparable for our age band (response rates among 65- to 84-year-olds were 79%, 68%, and 69% in the study waves initiated in 1994, 2007, and 2015, respectively) (25).

To enable us to study differences between nonoverlapping birth cohorts, we pooled data from the three waves and constructed three age bands: 66–72 years, 73–78 years, and 79–84 years. Within each age band we distinguished between birth cohorts (for 66–72 years this was 1921–1929, 1935–1942, and 1943–1949; for 73–78 years it was 1916–1922, 1928–1935, and 1937–1943; for 79–84 years it was 1910–1916, 1923–1929, and 1931–1937). Thus, we were able to compare grip strength within equivalent age bands by birth cohort to ensure that any differences in grip strength observed could be attributed to cohort rather than age differences. Additionally, to be able to compare cohort differences in grip strength when grouping the whole age range 66–84 years, and at the same time ensure nonoverlapping birth cohorts, we also ran comparisons with inclusion of only the study waves initiated in 1994 (born 1910–1929) and in 2015 (born 1931–1949). The total study population comprised 5,595 men and women (54% women) (Table 1).

**Table 1.** Number of Participants and Mean Age (*SD*) by Gender, Age Group, and Birth Cohort (BC), *N* = 5,595

Age and BC	Men, *n*	Age (*SD*)	Women, *n*	Age (*SD*)
66–72 years				
BC 1921–1929	820	68.9 (2.0)	999	68.8 (1.9)
BC 1935–1942	486	69.5 (1.8)	764	68.8 (2.0)
BC 1943–1949	242	69.5 (1.7)	125	69.7 (1.6)
73–78 years				
BC 1916–1922	226	74.0 (1.1)	282	73.9 (1.0)
BC 1928–1935	371	75.2 (1.7)	424	75.4 (1.7)
BC 1937–1943	158	75.4 (1.8)	121	75.1 (1.7)
79–84 years				
BC 1910–1916	10	80.5 (1.7)	19	81.3 (1.8)
BC 1923–1929	160	81.0 (1.7)	253	80.9 (1.6)
BC 1931–1937	79	81.2 (1.7)	56	81.3 (1.7)
Total	2,552		3,043	

### Assessment of Grip Strength

At all three study waves grip strength was assessed by trained health professionals using the same standardized protocol; grip strength of the non-dominant hand was measured in bar units using a Martin vigorimeter (26). A new device was used in 1994 and reused in 2007, with replacement device then used in 2015. The Martin vigorimeter comes with three balloon sizes and we used the largest and medium-sized balloons for men and women, respectively. Each participant was allowed two attempts, and the highest score registered was recorded and used in analyses. In the 2015 study wave, the majority of the sample had their grip strength measured with a Jamar Digital Dynamometer, while a randomly selected sub-set of 781 participants (22%) also had their grip strength measured with a Martin vigorimeter (with a break of 5–10 minutes between testing, and with the Martin vigorimeter used first). Only the values for the Martin vigorimeter were used in the current study to ensure fair comparison of grip strength across cohorts.

### Mediators

To address our second aim of investigating factors that may explain cohort differences in grip strength, we selected factors a priori which are known to be associated with grip strength (16,27,28) and whose population distribution was known to have changed between study waves. These were self-reports of education (stratified in three groups as primary (low), technical school/middle school/high school (middle), college/university (high)), daily smoking (current, former, never), leisure time physical activity (any participation: yes vs no), and measured height (cm) and weight (kg).

### Statistical Analyses

We compared grip strength across three nonoverlapping birth cohorts within each age band. Estimation of mean grip strength (with 95% confidence intervals) was modeled using linear regression, adjusted for age as a linear variable. Results were centered at the mean age within each age band (ie, 66–72 = 69; 73–78 = 75; 79–84 = 80). This was first done in sex-specific models, before they were combined, with sex then added to the model, and the grip strength estimate calculated with equal weights for men and women. To test for linear trend in grip strength across the three birth cohorts, within each age band, birth cohort (coded 0, 1, 2) was treated as a continuous term, adjusted for age and gender. To formally test if cohort trends differed by age band and gender, birth cohort by age band and birth cohort by gender interaction terms were added to the model.

**Figure 1.**
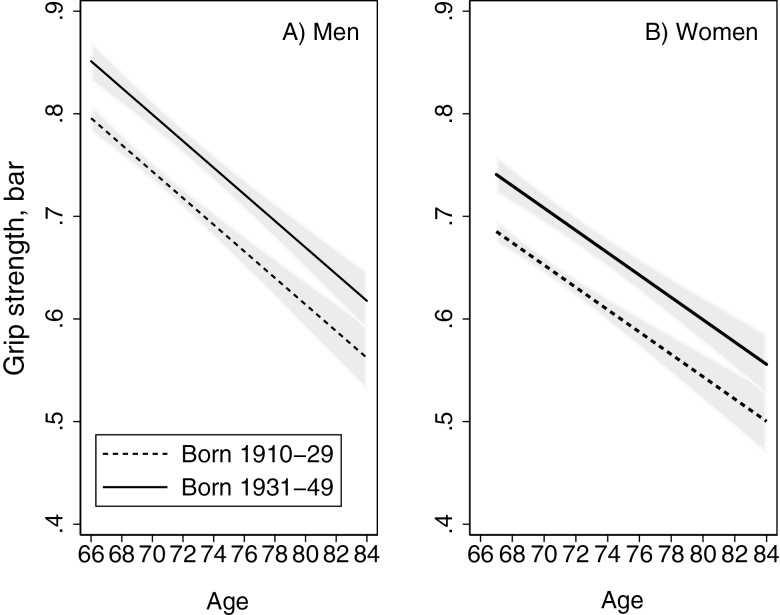
Grip strength (bar) with 95% confidence bands for two nonoverlapping birth cohorts, by age and gender. *N* = 3,137. Estimation was done using linear regression with the inclusion of birth cohort, gender, age and a gender × age interaction term. Plots are based on cross-sectional data.

In a second set of analyses using only the 1994 (birth cohort 1910–1929) and 2015 (birth cohort 1931–1949) study waves, the whole age range 66–84 years was investigated (*n* = 3,137). A regression model was fitted with grip strength as dependent variable with the inclusion of birth cohort, gender, age, and a gender by age interaction term (*p* = .06) (none of the other two-way or three-way interaction terms were statistically significant (*p* > .4) and therefore not included). Mean grip strength by age was predicted from this model and plotted (Figure 1). Finally, using this restricted study sample, a set of linear regression models were fitted adding mediators to the model. The sample was further restricted to those with non-missing values for all mediators (*n* = 3,032).

Tests of non-linearity across age were performed by modeling age as a restricted cubic spline with three knots with default knot locations. The model fit from this was no better than the simpler model with age as a linear variable (Likelihood-ratio test, *p*-value .89), and therefore we used the simpler model.

## Results

In all age groups, prevalence of higher educational levels increased in more recent born cohorts (Supplementary Table); for example, among males aged 66–72 years, the percentage with higher education increased from 15% among the cohort born in 1921–1929 birth cohort, to 30% among those born 1935–1942 and 38% in the cohort born 1943–1949. Prevalence of smoking among men decreased from 27% to 15% to 9%, mean height increased from 174 cm to 175 cm to 177 cm, while mean weight increased from 78 kg to 83 kg to 87 kg. Similar patterns were observed among women and in the older age bands. There was no clear pattern of cohort differences in leisure-time physical inactivity in the two youngest age bands, while in the oldest age band the prevalence declined across birth cohorts; 20% to 20% to 13% in men and 53% to 38% to 21% in women.

In all age bands, mean grip strength was higher in more recent born cohorts (Table 2). In the gender-combined analyses, cohort changes were similar across age groups (all age band by cohort interaction terms were nonsignificant). In the youngest age band (66–72 years), mean grip strength increased significantly in a step-wise pattern from 0.71 bar among those born in 1921–1929 to 0.72 bar among those born in 1935–1942, to 0.77 bar among those born in 1943–1949 (test for linear trend *p* < .001). The differences by birth cohort were similar among the two older age bands. There was no significant birth cohort by gender interaction within the two youngest age bands 66–72 years (*p* = .47) and 73–78 years (*p* = .99), while for the oldest age band (79–84 years) there was a significant birth cohort by gender interaction term (*p* = .03), suggesting a larger increase in grip strength across cohorts among men.

**Table 2.** Mean Grip Strength* (95% CI) in Bar by Gender, Age, and Birth Cohort (BC), *N* = 5,595

Age Band and BC	Men	Women	Both Genders Combined
66–72 years			
BC 1921–1929	0.76 (0.74, 0.77)	0.66 (0.65, 0.68)	0.71 (0.70, 0.72)
BC 1935–1942	0.77 (0.75, 0.79)	0.67 (0.65, 0.68)	0.72 (0.71, 0.73)
BC 1943–1949	0.82 (0.80, 0.85)	0.71 (0.68, 0.74)	0.77 (0.75, 0.79)
*p*-trend^†^	<.001	.067	<.001
Diff last-first BC, *p*-value	<.001	.009	<.001
73–78 years			
BC 1916–1922	0.67 (0.65, 0.70)	0.61 (0.59, 0.63)	0.64 (0.62, 0.66)
BC 1928–1935	0.70 (0.68, 0.71)	0.62 (0.60, 0.63)	0.66 (0.64, 0.67)
BC 1937–1943	0.72 (0.70, 0.75)	0.65 (0.62, 0.68)	0.68 (0.66, 0.70)
*p*-trend^†^	.009	.059	.001
Diff last-first BC, *p*-value	.008	.045	.001
79–84 years			
BC 1910–1916	-	0.55 (0.48, 0.62)	0.55 (0.49, 0.60)
BC 1923–1929	0.63 (0.61, 0.66)	0.58 (0.56, 0.60)	0.60 (0.62, 0.68)
BC 1931–1937	0.71 (0.67, 0.74)	0.59 (0.55, 0.63)	0.65 (0.62, 0.68)
*p*-trend^†^	<.001	.40	<.001
Diff last-first BC, *p*-value	-	.37	.001

*Notes*: *Grip strength was estimated using linear regression models centered at mean age within age bands (66–72 = 69; 73–78 = 75; 79–84 = 80).

^†^ *p*-trend was estimated using linear regression treating wave as a continuous 0, 1, 2 variable, adjusted by age (and further adjusted by gender, with equal weight for men and women, in the column where both genders are combined).

When examining the wider age band of 66–84 years, mean grip strength was higher in the most recent born birth cohort (1931–1949) compared to the oldest born cohort (1910–1929) in both sexes (Figure 1, Table 3). Furthermore, this advantage in the most recent birth cohort was similar across the whole age range from 66 to 84 years (as indicated by no evidence of a birth cohort by age interaction). None of the two-way or three-way interactions between birth cohort, gender and age were significant, so by excluding them, we could estimate the overall decline in grip strength by age, and the difference between birth cohorts collapsed over genders. In this model, grip strength declined by 0.06 bar per every 5 years (95% CI 0.05, 0.07). In comparison, the difference between birth cohorts was 0.06 (95% CI 0.04, 0.07). Thus, the difference between birth cohorts born 21 years apart corresponded to a 5-year difference in grip strength.

**Table 3.** Mean Difference in Grip Strength (Bar) Between Two (Nonoverlapping) Birth Cohorts, Among 66- to 84-Year-Olds^‡^

Birth Cohort	*N*	Model 1 “Age ^†^, gender”	Model 2 “Height”	Model 3 “Height, weight”	Model 4 “Educ.”	Model 5 “Height, weight, educ”	Model 6 “Smok.”	Model 7 “Inactive”	Model 8 “Fully adjusted”
1910–1929	2,326	Ref	Ref	Ref	Ref	Ref	Ref	Ref	Ref
1931–1949	706	0.056*	0.043*	0.039*	0.042*	0.029*	0.051*	0.055*	0.027*
Attenuation^‡^			23%	30%	25%	48%	9%	2%	52%

*Notes*: Estimated using linear regression. *N* = 3,032 in all models 1–8. Model 1: Adjusted by gender and age; Model 2: Model 1 + adjusted by height; Model 3: Model 1 + adjusted by height and weight; Model 4: Model 1 + education; Model 5: Model 1 + height, weight, and education; Model 6: Model 1 + daily smoking; Model 7: Model 1 + leisure time physical inactivity; Model 8: Fully adjusted.

^†^Mean age for birth cohort 1910–1929 was 70.1 (*SD* 3.0) and for 1931–1949 it was 73.4 (*SD* 4.6).

^‡^Percentage attenuation compared to model 1.

* *p* < 0.01.

Half of the increase in grip strength between the most recent and oldest born cohorts was mediated by higher educational level and greater height and weight, while cohort changes in smoking and physical inactivity had little impact (Table 3). The single most important mediator was education, which attenuated the cohort difference in grip strength by 25%. Height attenuated the cohort difference in grip strength by 23%, while height and weight combined attenuated the difference by 30%. In comparison, smoking attenuated the cohort difference in grip strength by only 9% and physical inactivity by 2%.

## Discussion

Our findings suggest improved grip strength in more recent birth cohorts of Norwegian men and women aged 66–84 years, which can be partly attributed to secular trends in education and growth.

This advantage in more recent birth cohorts was similar across the whole age range, which is suggestive of a difference at the intercept that is then carried forward with age, potentially relating to developmental differences. The most important mediators were education and height, which both have their origins in early life. Thus, cohort differences in grip strength in old age could be at least partly attributable to differences in exposures accumulating from early life onwards. Findings from birth cohorts including the MRC National Survey of Health and Development and Hertfordshire Cohort study have shown that early life factors have long-term associations with muscle strength; both higher birth weight and faster rates of growth in childhood and adolescence have been shown to be positively associated with grip strength in adulthood (29,30).

Published evidence on the association between education and grip strength is equivocal, and seems to vary between countries (27). However, in the Tromsø Study, those in higher educational groups have been found to have stronger grip (16). In accordance with our finding that cohort differences in grip strength may be at least partly explained by cohort differences in educational levels, improvements in education were found to be an important explanation of reductions in late-life disability in more recent born cohorts of Americans (31). As well as acting as a marker of early life influences, greater educational attainment is also associated with subsequent access to a range of beneficial resources in adulthood, such as financial security, health care, and healthy behaviors, and so could impact health and function in a number of different ways (32). That height was found to be a more important mediator than weight is perhaps to be expected given height is consistently found to be associated with grip strength whereas findings on the relationship between BMI and grip strength are equivocal (33). In an additional sensitivity analysis (not shown), weight was substituted for body mass index (kg/m^2^); results were similar and our main findings were unchanged.

Smoking and physical inactivity attenuated the cohort difference in grip strength to a lesser degree than education, height, and weight. This is possible because there are no such marked cohort differences in these variables; for example, even though there was a lower prevalence of current smoking in more recent birth cohorts, the prevalence of former smokers were similar across cohorts, suggesting similar levels of exposure to cigarette smoking. It is also possible that by measuring these factors at only one time point we did not capture all relevant lifetime variations in exposure. In addition, for physical activity we may not have captured variation in all relevant aspects of activity especially as one might expect opposing trends in occupational and leisure-time physical activity by birth cohort which if taken into account may have explained more of the difference. However, we are confident that the observed improvement in grip strength in more recent birth cohorts was not due to aggregation bias related to differences in age distributions within age bands, especially as mean age within age bands was actually higher in more recent birth cohorts.

Our findings of improved grip strength in more recent birth cohorts are at odds with those reported in Denmark, where grip strength was similar across birth cohorts (8). These differences could be due to country-specific processes, but could also be due to the inclusion of younger adults (66–84 years) born more recently in our study, compared with the Danish study (aged 93–95 years, birth years 1905 and 1915). This explanation might also apply to the contrasting findings in two other studies of older participants, one Swedish study (77–101 years) (9) and one Chinese study (80–105 years) (11). Both of these studies reported worse physical performance in more recent birth cohorts, but unfortunately these studies did not include grip strength measurements. Furthermore, in the Chinese study, the later born cohorts had significantly lower education and worse childhood conditions than earlier born cohorts, probably due to domestic wars (11).

It has been suggested that two opposing processes might drive changes in health and function across birth cohorts. First, more recently born cohorts might have benefitted more from advances in medical science and improved welfare and thereby reach older age in better health (denoted success-of-success). At the same time, these improvements might result in greater survival of less healthy adults, resulting in overall reduced population health at older age (denoted failure-of-success). In the Danish study, it was suggested that these two processes counterbalanced each other (8), while in our study it seems that success-of-success was the dominant process.

A strength of the current study is the high degree of generalizability due to recruitment from a general population. However, legal restrictions hamper detailed comparison of morbidity and mortality for attendees and non-attendees (34). In general, investigation of the two first waves, Tromsø 4 and 6, revealed differences in age and marital status between participants and non-attendees; non-attendees were younger and more likely to be single (34). Furthermore, whether these findings are generalizable outside Norway is unknown. A limitation is the small sample size for the oldest old (ie, age 79–84 years), especially men. For this reason, we could not estimate male grip strength in the oldest cohort in the oldest age band. Due to limited statistical power, we also had to rely on unconventional age bands. However, the same protocol and dynamometer type were used to assess grip strength in all birth cohorts. Thus, the improved grip strength in more recent birth cohorts is not likely to be attributable to changes in device or procedure. Unfortunately, we could not calibrate the devices since we did not have access to the old ones at the time of the most recent wave. Thus, we had to rely on factory calibration, and so cannot entirely rule out minor differences in measurement device. As in all studies with this observational design, falling response rates have been experienced in more recent study waves, and thereby the possibility of selection of a healthier population in more recent birth cohorts cannot be ruled out. However, response rates have remained high and while they did fall between the 1994 and 2007 waves, they stabilized in the 2015 wave and so if changes in response rates were explaining our findings one would have expected larger differences in grip strength between the first two waves but we observed larger changes between the latest two waves. Thus, we believe this bias is likely to be small in magnitude, and is unlikely to explain the grip strength improvement in more recent birth cohorts.

Our findings suggest improved grip strength in more recent birth cohorts of older Norwegian adults aged 66–84, which can be partly attributed to higher education and growth. The scale of these differences suggest that more recent born generations of 80-year-olds will have similar mean grip strength as 75-year-olds one generation earlier. This improved physical capability among older adults might impact on future forecasting of health and social care needs and costs.

## Funding

R.C. was supported by the Medical Research Council (Programme code MC_UU_12019/4). The funder had no role in study design, data collection, data analysis, data interpretation, or writing of the manuscript. B.H.S. had full access to all the data in the study and had final responsibility for the decision to submit for publication.

## Ethics approval and consent to participate

The Regional Committee of Research Ethics approved the study (2016/389).

## Conflict of Interest

None of the authors declare support from any organization for the submitted work; H.S. has received an unrestricted research grant from AstraZeneca in 2017, 65,000£ and a lecture fee of 700 £ from MSD, R.C. reports grants from UK Medical Research Council during the conduct of the study.

## Supplementary Material

Supplementary Table
