# Supplementary material for: Do More Recent Born Generations of Older Adults Have Stronger Grip? A Comparison of Three Cohorts of 66- to 84-Year-Olds in the Tromsø Study
Source: J Gerontol A Biol Sci Med Sci. 2018 Oct 11;74(4):528–33. doi: 10.1093/gerona/gly234 (PMC6417452; doi:10.1093/gerona/gly234)
Supplement: Supplementary Table [file gly234_suppl_supplementary_table.docx]

# Supplementary material

Supplementary Table . Distributions of potential mediators by gender, age and birth cohort (bc). N=5,595*.

|  | 66-72 years | | |  | 73-78 years | | |  | 79-84 years | | |
| --- | --- | --- | --- | --- | --- | --- | --- | --- | --- | --- | --- |
|  | bc 1921-29 | bc 1935-42 | bc 1943-49 |  | bc 1916-22 | bc 1928-35 | bc 1937-43 |  | bc 1910-16 | bc 1923-29 | bc 1931-37 |
| Men |  |  |  |  |  |  |  |  |  |  |  |
| Education, % |  |  |  |  |  |  |  |  |  |  |  |
| Low | 53 | 34 | 34 |  | 59 | 43 | 31 |  | 60 | 42 | 36 |
| Medium | 32 | 36 | 28 |  | 34 | 37 | 31 |  | 20 | 36 | 28 |
| High | 15 | 30 | 38 |  | 8 | 20 | 39 |  | 20 | 21 | 36 |
| Smoking, % |  |  |  |  |  |  |  |  |  |  |  |
| Current | 27 | 15 | 9 |  | 25 | 12 | 8 |  | 10 | 14 | 6 |
| Former | 56 | 61 | 59 |  | 54 | 66 | 58 |  | 60 | 69 | 59 |
| Never | 16 | 24 | 32 |  | 21 | 21 | 33 |  | 30 | 18 | 35 |
| Physically inactive, % | 11 | 17 | 14 |  | 16 | 22 | 17 |  | 20 | 20 | 13 |
| Height, cm, mean (sd) | 173.8 (6.7) | 174.7 (6.4) | 176.5 (6.3) |  | 173.1 (6.3) | 173.6 (6.8) | 174.4 (5.9) |  | 169.4 (9.4) | 172.7 (6.2) | 173.2 (6.2) |
| Weight, kg, mean (sd) | 78.0 (12.1) | 83.0 (12.0) | 86.8 (12.7) |  | 77.8 (12.8) | 81.1 (12.5) | 84.8 (12.5) |  | 66.8 (12.5) | 77.7 (11.1) | 79.1 (10.9) |
| BMI, (kg/m^2^), mean (sd) | 25.8 (3.5) | 27.2 (3.4) | 27.8 (3.8) |  | 25.9 (3.9) | 26.9 (3.6) | 27.9 (4.0) |  | 23.2 (3.4) | 26.0 (3.6) | 26.4 (3.2) |
| Women |  |  |  |  |  |  |  |  |  |  |  |
| Education, % |  |  |  |  |  |  |  |  |  |  |  |
| Low | 73 | 56 | 32 |  | 80 | 63 | 52 |  | 67 | 62 | 59 |
| Medium | 21 | 27 | 26 |  | 17 | 26 | 25 |  | 22 | 28 | 22 |
| High | 6 | 18 | 42 |  | 3 | 11 | 23 |  | 11 | 9 | 20 |
| Smoking, % |  |  |  |  |  |  |  |  |  |  |  |
| Current | 24 | 17 | 8 |  | 20 | 14 | 2 |  | 11 | 8 | 9 |
| Former | 30 | 38 | 45 |  | 23 | 34 | 47 |  | 11 | 36 | 37 |
| Never | 46 | 45 | 46 |  | 57 | 51 | 50 |  | 79 | 56 | 54 |
| Physically inactive, % | 18 | 18 | 15 |  | 25 | 27 | 19 |  | 53 | 38 | 21 |
| Height, mean (sd) | 160.1 (5.8) | 161.3 (6.2) | 162.7 (6.6) |  | 159.3 (6.5) | 159.6 (6.0) | 161.4 (6.2) |  | 158.1 (6.8) | 158.8 (5.7) | 157.9 (6.3) |
| Weight, kg, mean (sd) | 68.5 (12.4) | 69.9 (12.5) | 73.6 (14.9) |  | 67.9 (12.7) | 68.5 (12.0) | 71.7 (15.1) |  | 63.7 (12.5) | 68.8 (11.5) | 69.5 (14.6) |
| BMI, (kg/m^2^), mean (sd) | 26.7 (4.7) | 26.9 (4.6) | 27.9 (5.9) |  | 26.7 (4.5) | 26.9 (4.5) | 27.5 (5.5) |  | 25.4 (4.2) | 27.3 (4.3) | 27.9 (5.9) |

*Maximum available sample was N=5595, Number with missing values for each potential mediator were: education n=120 (2%); smoking n=77 (1%); physical activity n= 523 (9%), height and weight n=7 (0.1%).
